# Identification of a 119-bp Promoter of the Maize Sulfite Oxidase Gene (*ZmSO*) That Confers High-Level Gene Expression and ABA or Drought Inducibility in Transgenic Plants

**DOI:** 10.3390/ijms20133326

**Published:** 2019-07-06

**Authors:** Ziwei Xu, Meiping Wang, Ziting Guo, Xianfeng Zhu, Zongliang Xia

**Affiliations:** 1 College of Life Science, Henan Agricultural University, Zhengzhou 450002, China; 2 Library of Henan Agricultural University, Zhengzhou 450002, China; 3 School of Life Sciences, Henan University, Kaifeng 475004, China

**Keywords:** maize, sulfite oxidase, promoter, drought, transcriptional regulation

## Abstract

Drought adversely affects crop growth and yields. The cloning and characterization of drought- or abscisic acid (ABA)-inducible promoters is of great significance for their utilization in the genetic improvement of crop resistance. Our previous studies have shown that maize sulfite oxidase (SO) has a sulfite-oxidizing function and is involved in the drought stress response. However, the promoter of the maize *SO* gene has not yet been characterized. In this study, the promoter (*ZmSOPro*, 1194 bp upstream region of the translation initiation site) was isolated from the maize genome. The in-silico analysis of the *ZmSOPro* promoter identified several *cis*-elements responsive to the phytohormone ABA and drought stress such as ABA-responsive element (ABRE) and MYB binding site (MBS), besides a number of core *cis*-acting elements, such as TATA-box and CAAT-box. A 5′ RACE (rapid amplification of cDNA ends) assay identified an adenine residue as the transcription start site of the *ZmSO*. The *ZmSOPro* activity was detected by β-glucuronidase (GUS) staining at nearly all developmental stages and in most plant organs, except for the roots in transgenic *Arabidopsis*. Moreover, its activity was significantly induced by ABA and drought stress. The 5′-deletion mutant analysis of the *ZmSOPro* in tobacco plants revealed that a 119-bp fragment in the *ZmSOPro* (upstream of the transcription start site) is a minimal region, which is required for its high-level expression. Moreover, the minimal *ZmSOPro* was significantly activated by ABA or drought stress in transgenic plants. Further mutant analysis indicated that the MBS element in the minimal *ZmSOPro* region (119 bp upstream of the transcription start site) is responsible for ABA and drought-stress induced expression. These results improve our understanding of the transcriptional regulation mechanism of the *ZmSO* gene, and the characterized 119-bp promoter fragment could be an ideal candidate for drought-tolerant gene engineering in both monocot and dicot crops.

## 1. Introduction

Drought is a key environmental stress factor that impacts crop growth, development, and yield [1,2]. With global climate change, water deficit has become a severe threat to crop production [3]. The creation of drought-tolerant crops by transgenic technology is an effective strategy to combat this threat. To obtain efficient transgenic plants, an appropriate promoter that enables transgene expression at desired levels is very important [4,5,6]. Therefore, it is necessary for scientists to isolate various promoters and identify their activity characteristics.

At present, two types of promoters are currently utilized in plant gene engineering. One comprises constitutive promoters such as the *CaMV 35S* promoter and the maize *ubiquitin* promoter [4]; the other type comprises inducible promoters, such as the promoters of the genes *RD29A* (*responsive to dehydration 29A*) and *RD29B* [7]. The *CaMV 35S* promoter drives high-level gene expression in dicot plants, whereas the maize *ubiquitin* promoter is able to drive gene expression in monocot plants [8,9]. These constitutive promoters are capable of driving high-level transgene expression without specific temporal and spatial expression, which may result in physiological and metabolic dysfunction [10,11,12]. In addition, the inducible or tissue-specific promoters can modulate target gene expression given specific developmental or stress stimuli. Thus, some tissue-specific and stress-inducible promoters have been identified. For example, the *pF128* promoter was used in transgenic foxtail millet and maize plants to drive specific gene expression in the seeds [13]. The promoters of several drought- and salt-responsive genes, such as *RD29A*, *RD29B*, *Rab16*A (*responsive to abscisic acid*), *DREB2* (*dehydration responsive element-binding protein 2*), and *BADH* (*betaine aldehyde dehydrogenase*), have been used to drive target gene expression under abiotic stress conditions in transgenic *Arabidopsis*, rice, or wild wheat plants [13,14,15,16]. However, the majority of these specific promoters have weak ability to drive gene expression, which restricts their application in crops.

Several studies have indicated that the sulfur metabolism pathway is involved in environmental stress adaptation, such as drought and salinity, through balancing sulfur (S) metabolites in higher plants [17,18,19]. Sulfite oxidase (SO) participates in S metabolism by catalyzing the oxidation of toxic sulfite to sulfate in plants [20]. Previously, our laboratory isolated the full-length cDNA sequence of the *Zea mays SO* gene (*ZmSO*) and characterized its sulfite-dependent oxidizing activity in vitro and sulfite detoxifying function in planta [21]. Overexpression of the *ZmSO* in tobacco improves drought tolerance by affecting stomatal regulation, the glutathione (GSH)-dependent antioxidant system, and S-metabolism-related gene expression [22]. However, the promoter region of this gene has not yet been characterized. In the present study, the activity of the 1194 bp promoter region upstream of translation start site of *ZmSO* was functionally characterized in transgenic *Arabidopsis* and tobacco through deletion analysis. Ultimately, a 119 bp fragment with high promoter activity that enables drought- and abscisic acid (ABA)-inducible gene expression were identified. This study provides novel insights into the understanding of the S metabolic pathway and promoter resources for engineering drought-tolerant crops.

## 2. Results

### 2.1. Isolation and Sequence Analysis of the ZmSO Promoter

**Figure 1:**
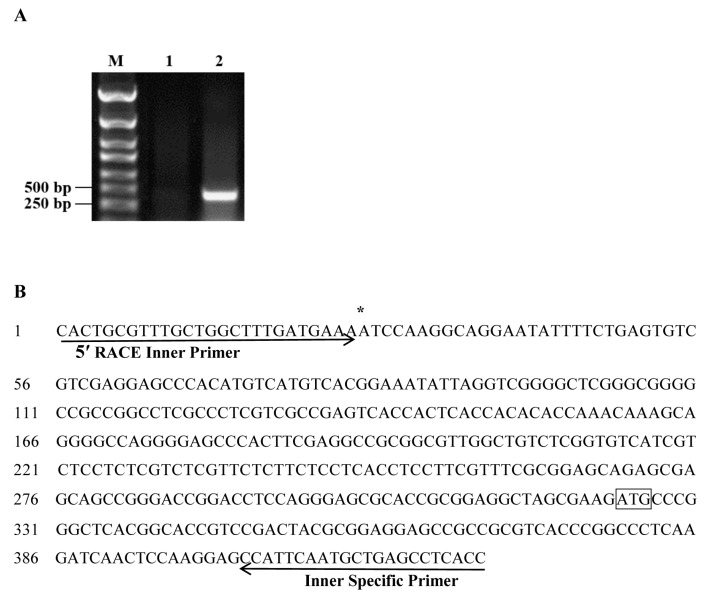
Identification of *ZmSO* transcription start site by 5′ RACE (rapid amplification of cDNA ends) assay. (**A**) 5′ RACE-PCR results. M: DNA marker DL2000; 1: outer 5′ RACE-PCR products; 2: inner 5′ RACE-PCR products. (**B**) Sequence alignment. The arrows represent the primers. The asterisk “*” represents the transcription start site. The black rectangle represents the translation initiation site.

The 1194 bp promoter sequence of *ZmSO* upstream of the translation initiation site (ATG) was isolated by PCR from maize genomic DNA. To identify the transcription start site of the *ZmSO* gene, we conducted a 5′ RACE (rapid amplification of cDNA ends) assay. The amplified fragment (approximately 400 bp in length) was purified and sequenced to determine the 5′ ends of the product (Figure 1A). Sequence analysis showed that the *ZmSO* transcription start site is an adenine (A) base flanked by thymine (T) and cytosine (C), and is located 297 bp upstream of the ATG translation initiation site (Figure 1B). This was consistent with a previous finding that the presence of an adenine in the transcription start site is flanked by pyrimidine bases in most plant genes [23].

**Figure 2:**
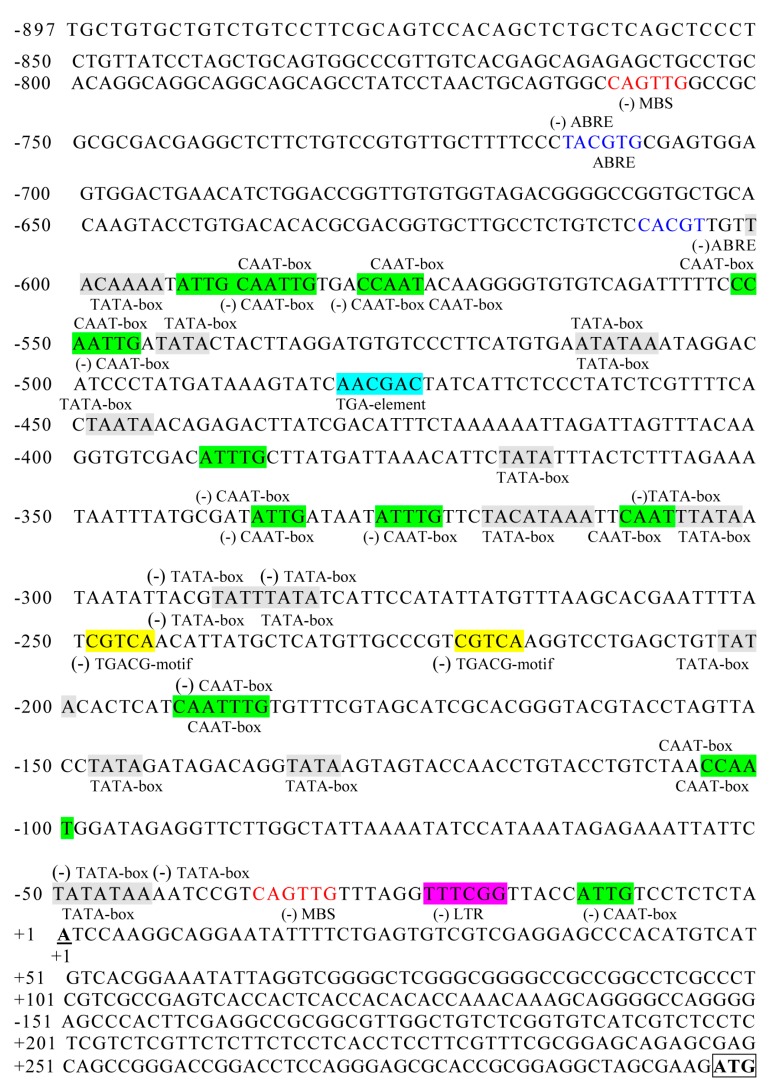
Nucleotide sequence of the *ZmSO* promoter. The transcription start site “A” of *ZmSO* is numbered as +1. The putative CAAT and TATA boxes are highlighted in green and grey, respectively. The abscisic acid (ABA)-responsive elements (ABREs) and MYB binding sites (MBSs) are marked in blue and red letters, respectively. The TGACG-motif and the TGA-element are highlighted in yellow and indigo, respectively. The low-temperature-responsive element (LTR) is highlighted in pink.

The putative *cis*-acting elements in the *ZmSO* promoter sequence were analyzed by PlantCARE. As shown in Figure 2 and Table S1, eight types of known *cis*-acting elements were indicated in total in the 1194 bp promoter sequence (the transcription start site A is numbered as +1). Besides a number of core *cis*-acting elements, such as 20 TATA-box and 17 CAAT-box elements, some *cis*-acting elements putatively involved in hormone and environmental stimuli were found. These elements include three ABA responsive elements (ABREs), two methyl jasmonate (MeJA) responsive elements (TGACG-motif), two G-box elements involved in light responsiveness, two MYB binding sites (MBSs) involved in drought induction, one low-temperature-responsive element (LTR), and one TGA element involved in auxin response (Figure 2 and Table S1).

### 2.2. Promoter Activity of ZmSO in Transgenic Arabidopsis

**Figure 3:**
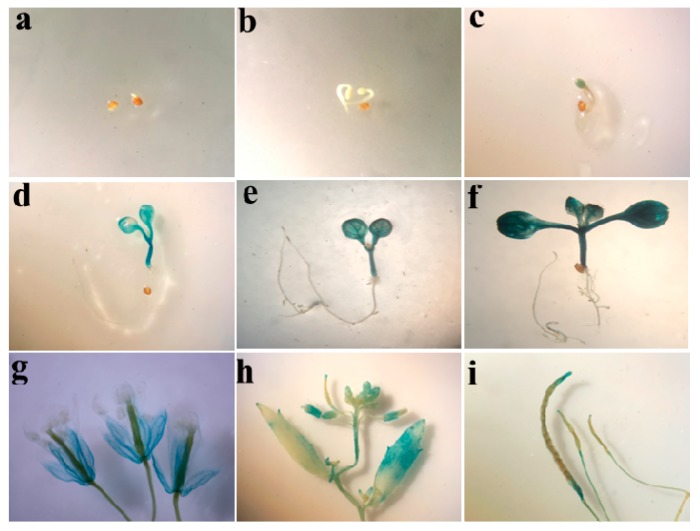
β-glucuronidase (GUS) staining of *ZmSOpro:GUS* transgenic *Arabidopsis* from different growth stages: (**a**) one-day-old germinating seedling; (**b**) two-day-old seedling; (**c**) three-day-old seedling; (**d**) four-day-old seedling; (**e**) six-day-old seedling; (**f**) ten-day-old seedling; (**g**) inflorescences from 7-week-old plants; (**h**) immature siliques from 8-week-old plants; (**i**) mature siliques from 10-week-old plants. Experiments were repeated at least two times with similar results. In each experiment, three independent transgenic lines were observed for promoter activity.

To comprehensively analyze activity of the *ZmSO* promoter, the 1194-bp promoter sequence of *ZmSO* was fused with the β-glucuronidase (*GUSi*) gene to generate transgenic *ZmSOpro:GUS Arabidopsis* plants, which were used to examine its expression in different developmental stages. Generally, GUS expression was detected by histochemical staining at most developmental stages, from seed germination to flowering, even maturing (Figure 3). In germinating seeds, no GUS activity was detected (Figure 3a,b). GUS activity was first detected in 3-day-old germinated seeds with weaker GUS staining in the emerging radicles (Figure 3c). Higher GUS expression was detected in hypocotyls of 4- and 6-day-old seedlings (Figure 3d,e). At ten days, GUS activity was detected throughout the whole plant, except for the roots (Figure 3f). In six- or eight-week-old plants, strong GUS staining was clearly observed in inflorescences and mature siliques (Figure 3g–i).

### 2.3. Changes of the ZmSO Promoter Activity in Arabidopsis in Response to ABA and Drought Stress

To further understand transcriptional responses of the *ZmSOpro:GUS* transgenic *Arabidopsis* to ABA and drought stress, two-week-old transgenic seedlings from four independent lines (SOP0-3, -4, -5, and -9) were treated with 50 µM ABA or 10% (*w*/*v*) polyethylene glycol(PEG) 6000 for 6 h, or transferred onto filter papers to induce dehydration stress for 2 h. To show the effectiveness of the treatments, we examined the expression of the desiccation-induced gene *RD29A* [7] and the drought- or osmotic-responsive gene *DREB2A* [24] in transgenic *Arabidopsis* expressing *ZmSOpro:GUS* or *CaMV* 35S under dehydration and osmotic stresses by qPCR. As we expected, compared to their corresponding controls, significantly increased expression of both stress-inducible marker genes were observed under dehydration or PEG-induced osmotic stress, both in *ZmSOpro:GUS* and *CaMV* 35S transgenic *Arabidopsis* plants (Figure S1A,B).

**Figure 4:**
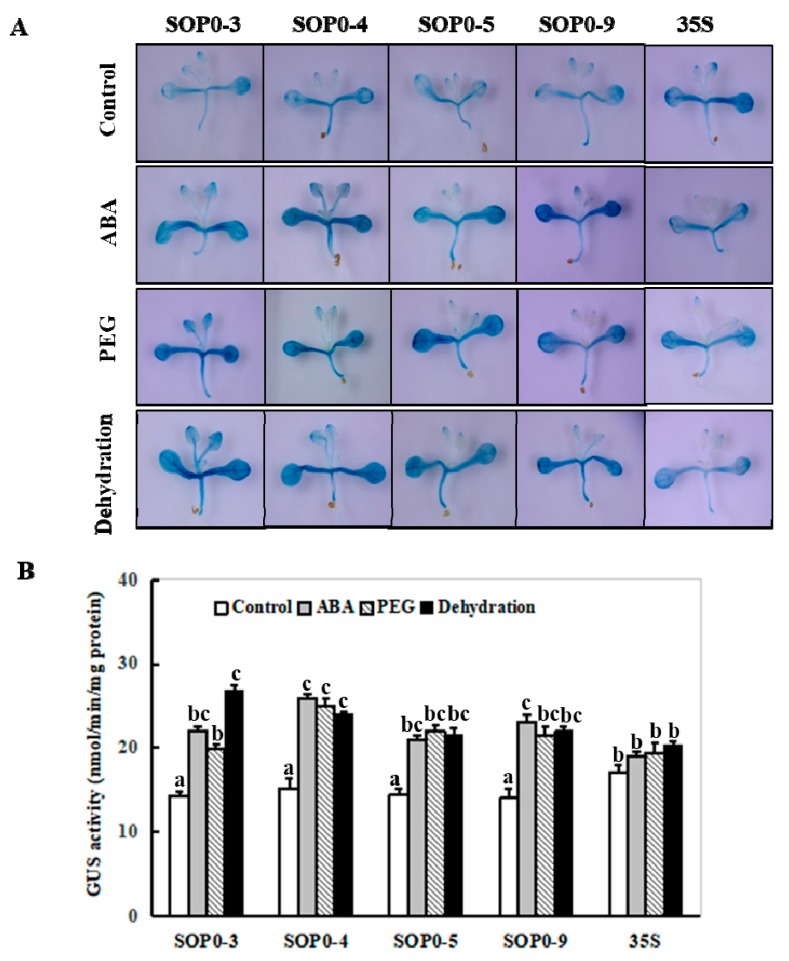
Histochemical and fluorescent quantitative analysis of transgenic *Arabidopsis* expressing *ZmSOpro:GUS* under normal, ABA, osmotic and dehydration conditions. (**A**) GUS histochemical staining; (**B**) GUS fluorescent quantitative analysis. Two-week-old *ZmSOpro:GUS* (SOP0-3, -4, -5, and -9), along with *CaMV* 35S transgenic *Arabidopsis* were incubated in liquid MS medium supplemented with 50 µM ABA or 10% (*w*/*v*) PEG 6000 to realize ABA or osmotic stress for 6 h, and the seedlings were transferred onto filter papers to induce dehydration stress for 2 h. The plants grown in the liquid MS medium were treated as controls. In (**B**), values represent the mean ± standard deviation from five individual plants for each line. Different lower-case letters above the bars indicate significant differences at *p* < 0.05.

Histochemical detection of the *ZmSO* promoter activity was performed with GUS staining in these stressed seedlings along with controls. As shown in Figure 4A, all these four transgenic *Arabidopsis* lines showed much higher GUS staining than their corresponding controls upon ABA, PEG treatment, or dehydration stress. (Figure 4A). This can be seen from the lower two leaves of these transgenic lines under stress conditions (Figure 4A). No significant differences in GUS staining intensity were observed among these *ZmSOpro:GUS* transgenic lines under control conditions (Figure 4A). Quantitative determination of the GUS staining under these stresses further demonstrated that compared to their corresponding controls, these transgenic lines increased significantly in GUS staining intensity (nearly 60% increase for ABA, 50% increase for PEG, and 65% increase for dehydration, on average) under ABA, PEG, or dehydration stress (Figure 4B). Noticeably, the *35S:GUS* transgenic line in GUS staining intensity showed no significant induction under these stresses compared to its corresponding control (Figure 4A,B).

### 2.4. Identification of the Minimal Region of the ZmSO Promoter Required for Its High-Level Expression

**Figure 5:**
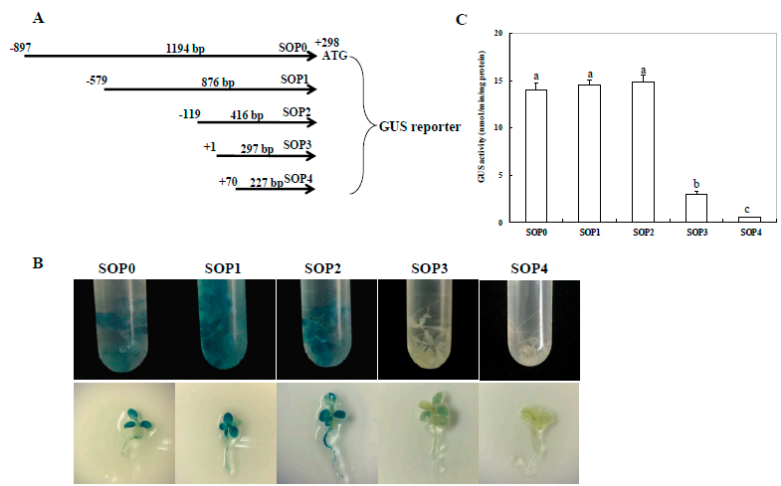
Histochemical and fluorescent quantitative analysis of transient expression of full-length and truncated *ZmSOpro:GUS* in tobacco seedlings. (**A**) The constructs of the truncated fragments of *ZmSOpro* fused with GUS. A series of 5′-deleted fragments (SOP0, SOP1, SOP2, SOP3, and SOP4) of *ZmSOpro* were placed upstream of the GUS reporter gene. The numbers indicate the nucleotide position from the transcription start site ATC (the transcription start site A is numbered as +1 and the translation initiate site ATG is numbered as +298). (**B**) GUS histochemical staining of transient expression of full-length and truncated *ZmSOpro:GUS* in tobacco seedlings. Sterile cultured tobacco seedlings at the 3- or 4-leaf stage were infiltrated by *Agrobacterium tumefaciens* harboring full-length or each truncated *ZmSOpro:GUS* construct, and then about six–eight seedlings for each construct were used for GUS histochemical staining. (**C**) GUS quantitative analysis of transient expression of full-length and truncated *ZmSOpro:GUS* in tobacco seedlings. Values represent the mean ± standard deviation from five individual plants for each construct. Different lower-case letters above the bars indicate significant differences at *p* < 0.05.

To identify the minimal region of the *ZmSO* promoter required for high-level expression, a series of deletion constructs (5′-deleted fragments) fused to the *GUS* gene were sequentially constructed (Figure 5A) and transformed into tobacco by the seedling agro-infiltration method. Promoter activities of the full-length *ZmSOpro* and its truncated versions in tobacco seedlings were determined by GUS staining. First, promoter activities between SOP1 (876 bp; −579 to +297), SOP2 (416 bp; −119 to +297), and SOP0 (1194 bp; −897 to +297) were compared (the transcription start site A is numbered as +1). As shown in Figure 5B, both truncated versions and the full-length *ZmSOpro* seedlings showed strong GUS staining intensity without significant differences (Figure 5B,C), suggesting that the 5′-terminal region (778 bp in length) in the *ZmSOpro* is not required for its high-level expression. Then, one deletion mutant SOP3 (297 bp; +1 to +297) was constructed and its promoter activity was investigated in tobacco seedlings. Surprisingly, this type of mutant tobacco seedling showed weak GUS staining intensity when compared to the full-length *ZmSOpro* (Figure 5B). GUS quantitative determination further demonstrated that the SOP3 mutant had more than 60% decreases in GUS intensity compared with the full-length *ZmSOpro* seedlings (Figure 5C), indicating that the 119-bp region (−119 to −1) in the SOP2 is necessary for its high-level expression. Finally, the SOP4 mutant (227 bp; +70 to +297) was constructed and displayed no GUS staining, implying that the last 227-bp sequence had no gene-driving ability (Figure 5B). Together, these results demonstrate that the 119-bp fragment in the *ZmSO* promoter is a minimal region, which is required for its high- level expression.

### 2.5. Dynamic Changes of the Minimal ZmSO Promoter Activity in Arabidopsis in Response to ABA or Drought Stress

**Figure 6:**
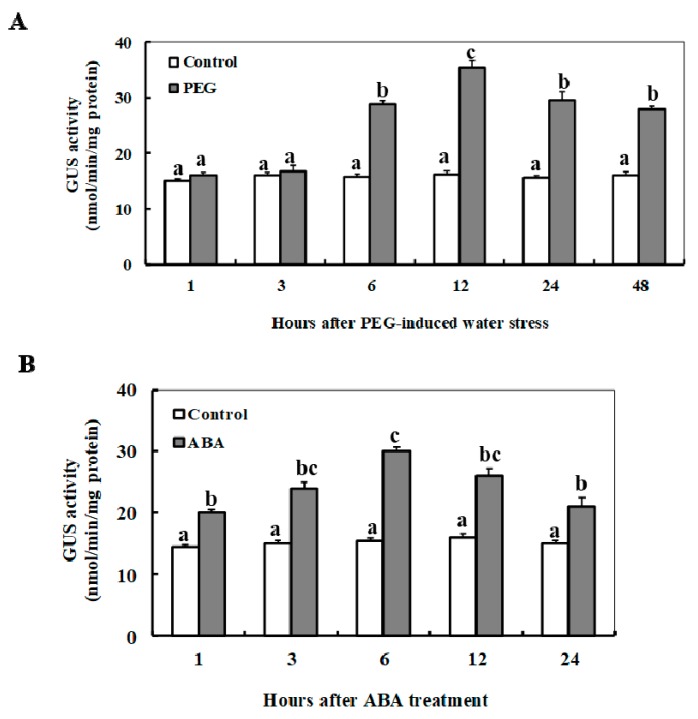
Changes of GUS activities in transgenic *Arabidopsis* expressing *ZmSOpro-P2:GUS* in response to ABA and PEG treatments. (**A**) GUS fluorescent quantitative analysis of *ZmSOpro-P2:GUS* transgenic *Arabidopsis* during PEG treatment. (**B**) GUS fluorescent quantitative analysis of *ZmSOpro-P2:GUS* transgenic *Arabidopsis* during ABA treatment. In both (**A**) and (**B**), two-week-old *ZmSOpro-P2:GUS* transgenic *Arabidopsis* plants were incubated in the liquid MS medium supplemented with 10% (*w*/*v*) PEG 6000 or 50 µM ABA to realize osmotic stress or ABA for 1, 3, 6, 12, 24, and 48 h. The plants grown in the liquid MS medium were treated as controls. In these figures, values represent the mean ± standard deviation from three independent transgenic lines and five individual plants for each line. Different lower-case letters above the bars indicate significant differences at *p* < 0.05.

Dynamic transcriptional responses of the minimal *ZmSO* promoter (*ZmSOpro*_min_) in transgenic *Arabidopsis* to ABA or drought stress were examined by quantitative determination of GUS activity (Figure 6A,B). Under PEG-induced water stress, the activity of the *ZmSOpro*_min_ was significantly up-regulated from 6 to 48 h of the stress period with a peak at 12 h (120% increase) (Figure 6A). Under ABA treatment, the *ZmSOpro*_min_ activity was promptly activated at 1 h of ABA treatment, and then maintained higher levels during 24 h of the stress period with a peak at 6 h (95% increase) (Figure 6B). These results indicated that the minimal *ZmSO* promoter was significantly activated by ABA or drought stress at early stage.

### 2.6. The MBS Element in the ZmSO Promoter Region between −119 and −1 Is Responsible for ABA- and Drought-Stress-Induced Expression

**Figure 7:**
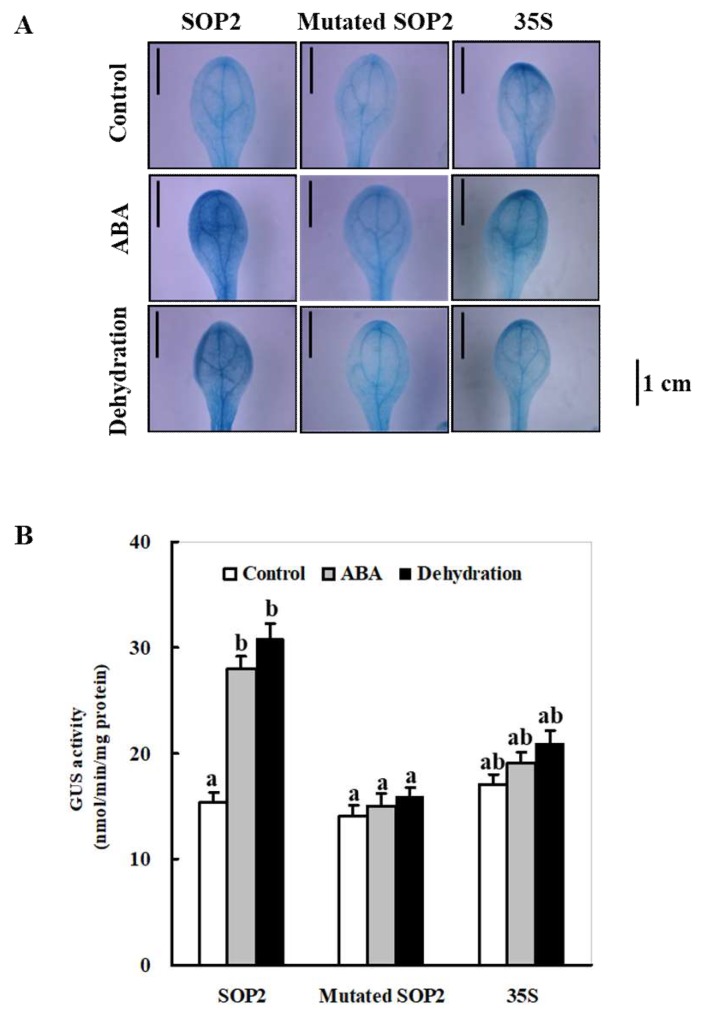
Histochemical and fluorescent quantitative analysis of transgenic *Arabidopsis* expressing wild-type and mutated *ZmSOpro-P2:GUS* under normal, ABA, and dehydration conditions. (**A**) GUS histochemical staining; (**B**) GUS fluorescent quantitative analysis. Two-week-old wild-type and mutated (point mutations in the MYB binding site (MBS) element) *ZmSOpro-P2:GUS*, along with *CaMV* 35S transgenic *Arabidopsis* plants were incubated in the liquid MS medium supplemented with 50 µM ABA for 6 h, and the seedlings were transferred onto filter papers to induce dehydration stress for 2 h. The plants grown in the liquid MS medium were treated as controls. Lower leaves were sampled for GUS staining and quantitative analysis. Bars =1 cm. In (**B**), values represent the mean ± standard deviation from five individual plants for each line. Different lower-case letters above the bars indicate significant differences at *p* < 0.05.

To further confirm whether the MBS element in the *ZmSO* promoter region between −119 and −1 is responsible for ABA and drought-stress-induced expression, the *ZmSOpro*_min_ (*SOP2*) with a point-mutated MBS element fused to the *GUS* gene was constructed and transformed into *Arabidopsis* by the floral dip method. The promoter activity of the mutated *SOP2*, together with the wild-type *SOP2* and the *CaMV 35S*, in transgenic *Arabidopsis* plants was determined by GUS staining under control, ABA, and dehydration conditions. As shown in Figure 7A, the wild-type *SOP2 Arabidopsis* leaves showed much higher GUS staining than their corresponding controls upon ABA or dehydration stress (Figure 7A). In contrast, leaves from the mutated *SOP2* or the *CaMV 35S* transgenic plants did not display clear changes in GUS staining intensity upon either ABA or dehydration stress (Figure 7A). This demonstrated that mutations in the MBS element of the *SOP2* largely attenuated its ABA- or dehydration-inducible expression. Quantitative determination of the GUS activities further verified this observation (Figure 7B). Together, these results demonstrate that the MBS element in the 119-bp *ZmSOpro*_min_ region is responsible for ABA- and drought-stress-induced expression.

## 3. Discussion

Our previous studies have shown that *ZmSO* detoxifies excess sulfite into sulfate and confers drought tolerance by affecting the sulfur metabolic pathway [21,22]. However, the mechanism underlying its transcriptional regulation has not been characterized. In this study, we characterized its promoter with ABA- or drought-inducible expression and identified a 119-bp minimal promoter region which might have good potential for application in plant genetic engineering.

Promoter activity analyses revealed that *ZmSO* was expressed at nearly all developmental stages, from seed germination to flowering, even in mature transgenic *Arabidopsis* (Figure 3), indicating that this gene may be constitutively expressed during both vegetative and reproductive growth. Besides leaves, higher activity levels of the *ZmSOpro:GUS* were observed in flowers and mature siliques, suggesting that the SO-dependent sulfite oxidation pathway may be involved in sulfur-containing protein biosynthesis during flower and seed development in plants. Interestingly, no activity of the *ZmSOpro:GUS* was detected in roots. In accordance with this observation, *ZmSO* transcripts were found to be higher in leaves and immature embryos, but very low in roots [22], indicating that *ZmSO* may play a role in the aerial parts of plants.

In this study, in-silico analysis of the *ZmSO* promoter region identified several consensus *cis*-acting elements related to stress responses, such as abscisic acid (ABA)-responsive elements (ABREs) and MYB binding sites (MBSs) involved in drought induction (Figure 2 and Table S1). GUS staining further confirmed that the *ZmSOpro* activity was detected in the guard cells of mature *Arabidopsis* leaves and up-regulated by ABA and drought stress (Data not shown). These results indicate that *ZmSO* might be involved in ABA signaling and drought stress responses in plants. In support of this hypothesis, our recent results showed that *ZmSO* was rapidly induced by drought and that *ZmSO* overexpression improved tolerance to drought stress [22]. Moreover, *ZmSO* positively regulated stomatal closure, which may reduce transpiration water loss in response to drought stress ([22]). Further work will be interesting to examine whether *ZmSO* is involved in the drought stress response in the ABA-dependent pathway.

The analysis of 5′-deleted mutants of *ZmSOpro* revealed that a 119 bp fragment (upstream of the transcription start site) is the minimal promoter region, which is required for its high-level expression in plants (Figure 5). Moreover, the minimal *ZmSO* promoter was significantly activated by ABA or drought stress (Figure 6). Noticeably, the 119-bp segment (−119 to −1) in the SOP2 mutant was necessary for its high-level expression (Figure 5B). However, several types of *cis*-acting elements, including three CAAT-box, three TATA-box, and one drought-inducible MYB-binding site (MBS) were found in the 119 bp region (Figure 2 and Table S1). Further mutant analysis showed that the MBS element in the 119-bp *ZmSOpro*_min_ region was responsible for ABA or drought-stress-induced expression (Figure 7). Generally, the CAAT-box is a common *cis*-acting element with enhancer activity in promoters, and the TATA-box is a core promoter element that initiates gene transcription. The transcription factor MYB binds the key element MBS in response to drought or ABA stress. However, there is no ABRE element in the *ZmSOpro*_min_ region. Thus, it could be speculated that the 119 bp segment which contains these key *cis*-acting elements might be crucial for *ZmSO*’s high-level expression and its response to ABA or drought stress. Generally, most *cis-*acting elements are located upstream of TATA-box motifs. However, in our study the putative MBS was located downstream of several putative TATA-box motifs (Figure 2). In agreement with our findings, Bhuria et al. (2016) found that in the promoter of the *Arabidopsis* universal stress protein (USP) gene *AtUSP*, the *cis*-acting elements such as the W-box (TTGAC at the −5 site from ATG) for pathogen- and salicylic acid (SA)- inducible expression, and MYB1 consensus (at −39 site from ATG) for ABA- and drought-responsive sites are all located downstream of the TATA-box motif (at the −95 site from ATG) [25]. Therefore, it would be meaningful to identify unknown regulatory proteins that can bind to these *cis*-elements in the minimal promoter region in order to improve our understanding of *ZmSO*’s ABA- or drought-stress-responsive mechanisms in future work.

Promoters can initiate gene transcription and regulate gene expression both temporally and spatially. It is essential to enable transgene expression at desired levels using an efficient promoter in gene engineering [4]. Thereby, the development of new promoters with tissue specificity or stress inducibility is attracting great attention from scientists in plant genetic engineering [26]. The utilization of inducible promoters can not only prevent unnecessary gene expression caused by constitutive promoters, but also reduce transgenic safety risks caused by virus-derived promoters such as *CaMV 35S* [27]. Here, we identified a 119 bp core fragment of *ZmSO* promoter with high-level expression and ABA/drought inducibility. Thus, we believe that the minimal fragment of the *ZmSO* promoter could have potential application in plant gene engineering based on two aspects. First of all, the core fragment of *ZmSO* promoter is only 119 bp in length and displays ABA/drought-inducible expression, which is useful for avoiding the repetitive use of the constitutive promoters and reducing the vector size for efficiently driving expression of transgenes under drought stress. More importantly, the 119 bp core fragment of the *ZmSO* promoter from maize (monocot) confers high-level and stress-inducible gene expression in both transgenic *Arabidopsis* (dicot) and tobacco (dicot) plants. As we know, *ubiquitin* promoters are more capable of driving transgene expression than the *CaMV 35S* promoter in monocots [6,28,29]. On the contrary, some *ubiquitin* promoters from monocots fail to drive transgene expression in dicots [30]. The minimal core promoter of *ZmSO* identified in our study will be more useful for drought-tolerant molecular breeding in both monocot and dicot crops. Further work is needed to characterize the activity of the 119 bp core promoter in maize.

Taken together, a 119 bp fragment of the *ZmSO* identified in this study could drive high-level transgene expression in an ABA/drought-inducible manner in transgenic plants, which might be utilized as a novel drought-inducible promoter system in plant genetic engineering. In future work, it will be meaningful to identify new transcription factors that regulate the minimal core promoter to develop an understanding of the ABA/drought- responsive mechanism of *ZmSO* and to accelerate the application of the inducible promoter system in the molecular breeding of maize.

## 4. Materials and Methods

### 4.1. Cloning of the ZmSO Promoter

To isolate the *ZmSO* promoter sequence, primers were screened from the 2 kb 5′ flanking region of the start codon (ATG) of *ZmSO* (Accession no. FJ436404). Consequently, the 1194 bp fragment upstream of the ATG of *ZmSO* was obtained by PCR from maize genomic DNA with primer pairs ZmSOPF and ZmSOPR (Table S2), and was cloned into a pGEM-T easy vector to confirm its fidelity by sequencing, as described previously [31].

### 4.2. Identification of the ZmSO Gene Transcription Start Site

To identify the transcription start site of *ZmSO*, 5′ RACE was performed using the FirstChoice^®^RLM-RACE Kit (Thermo Fisher Scientific, Waltham, MA, USA). In brief, total RNA was treated with calf intestinal phosphatase (CIP) and tobacco acid pyrophosphatase (TAP). Then, a 45-base RNA adapter was ligated to the RNAs. Following this, a random-primed reverse transcription reaction and nested PCR were used to amplify the 5′ end of a specific transcript. Finally, two gene-specific reverse primers (outer-specific and inner-specific primers, see Table S2) were designed to amplify the specific transcript with the forward primers provided by the kit. The amplified PCR products were sequenced.

### 4.3. Bioinformatic Analysis of the ZmSO Promoter Sequence

The *cis*-regulatory elements of 1194 bp *ZmSO* promoter sequence were analyzed using the online software PlantCARE [32].

### 4.4. Construction of Plasmids Harboring Full-Length and Mutant Promoters Fused to GUS

The full-length (SOP0) and four 5′-deleted fragments (SOP1-SOP4) with different sizes (1194, 876, 416, 297, and 227 bp; Figure 5A) were amplified by PCR with the corresponding primers (Table S2). These truncated fragments were subsequently sub-cloned into the pCAMBIA1381 vector containing a *GUS* reporter with *Eco*RI/*Hin*dIII restriction sites and confirmed by sequencing. To test the role of the MBS element in the SOP2 activity, the SOP2 fragment with a point-mutated MBS element was also amplified by PCR with the corresponding primers (Table S2) and sub-cloned into the same vector as described above.

### 4.5. Agrobacterium-Mediated Transformation of Arabidopsis Plants

Five-week-old *Arabidopsis thaliana* (Col-0) plants grown in our growth room under normal conditions (22 °C, 16 h/8 h photoperiod, 200 μmol m^−2^ s^−1^ light intensity) were used for *Agrobacterium-*mediated transformation using floral dip method [33]. The transformed *Arabidopsis* plants were maintained to grow for setting seeds. Then, seeds of the T_0_ generation were screened on MS medium supplemented with hygromycin (20 mg/L). The positive transgenes (T1 plants) that were hygromycin resistant and verified by PCR were transferred to pots with nutrient soils to grow to maturity for setting seeds (T_2_ seeds). T_2_ seeds were germinated on hygromycin-selective medium again, and the one-copy lines were identified by examining the segregation ratio (3:1) of the hygromycin-selectable marker. Each one-copy line was maintained to set seeds until the T_3_ generation. The homozygous T_3_ transgenic *Arabidopsis* lines harboring pCAMBIA1381-SOP0, along with the positive control (pCAMBIA1381-35S) were used for ABA or stress treatments and GUS staining assays.

### 4.6. Histochemical and Quantitative GUS Assays

Histochemical GUS staining was performed as described previously [34,35,36]. In brief, *Arabidopsis* or tobacco seedlings were incubated at 37 °C for 12 h in GUS staining buffer containing 1 mM 5-bromo-4-chloro-3-indolyl-β-D-glucuronic acid (X-Gluc), 100 mM sodium phosphate buffer (pH 7.0), 0.5 mM potassium ferrocyanide, 0.5 mM potassium ferricyanide, 10 mM EDTA, and 0.1% Triton X-100. Then, the seedlings were decolorized in 70% ethanol and photographed using a stereomicroscope with a digital camera (Nikon, Osaka, Japan).

Quantitative determination of GUS activity by fluorometric assay was performed according to the method of Jefferson et al. [35]. Briefly, plant tissues were homogenized in a pre-cooling extraction solution (0.1% sodium lauryl sarcosine, 100 mM sodium phosphate (pH 7.0), 10 mM EDTA, 10 mM DTT, and 0.1% Triton X-100). After centrifugation, GUS activity was detected at 37 °C with the buffer containing 1 mM 4-methylumbelliferyl-β-glucuronide (4-MUG). The fluorescence was quantified using a fluorescence spectrophotometer (HITACHI F-4600, Tokyo, Japan). Protein concentration was determined as described previously [37,38]. The GUS activity was normalized with 4-MU standards and expressed as nmol MU per minute per mg protein. Values represent the mean ± standard deviation from three independent transgenic lines and five individual plants for each line.

### 4.7. ABA, Dehydration, and Osmotic Stress Treatments

Sterile seeds of *Arabidopsis* or tobacco were germinated and grown in plates containing MS solid medium in a growth chamber at 23 °C with a cycle of 16 h light/8 h dark. After two weeks, *Arabidopsis* or tobacco seedlings were incubated in liquid MS medium supplemented with 50 µM ABA or 10% (*w*/*v*) PEG 6000 to realize ABA or osmotic stress, and the seedlings were transferred onto filter papers to induce dehydration stress for 2 h, as described previously [39,40,41]. For transgenic *Arabidopsis*, the period of ABA and PEG lasted 6 h. For *A. tumefaciens*-infiltrated tobacco, the period of PEG and ABA treatments lasted 48 h and 24 h, respectively. The whole plants were sampled at the indicated time for GUS histochemical staining or GUS fluorometric assays. For GUS staining of guard cells, 2-week-old *ZmSOpro:GUS* transgenic *Arabidopsis* seedlings were treated by dehydration for 2 h or by 5 µM ABA for 3 h. Then, epidermal peels of leaves were stripped to perform GUS histochemical staining. Following this, guard cells were observed with a microscope (Nikon, Osaka, Japan) as described previously [42,43]. All the assays were conducted three times with independent samples.

### 4.8. Transient GUS Expression Assays in Tobacco Seedlings

The pCAMBIA1381 and recombinant plasmids SOP0–P4 were introduced into the *Agrobacterium tumefaciens* GV3101 using a freeze–thaw method. The *A. tumefaciens* harboring each construct was oscillatorily cultured in bottles containing YEP liquid medium supplemented with kanamycin (50 mg/L) and rifampicin (50 mg/L) at 28 °C. After 24 h, the cultured bacteria were collected by centrifugation and re-suspended in the transformation buffer containing 10 mM MgCl_2_, 10 mM MES, and 0.1 mM acetosyringone to an OD_600_ of 0.8–1.0 for tobacco transformation. Sterile cultured tobacco seedlings at the 3- or 4-leaf stage were submerged into the transformation solution for 2 h, and then the infiltrated seedlings were co-cultured in a moist chamber for 48 h. Finally, the infiltrated seedlings harboring different constructs were washed three times with ddH_2_O and used for histochemical GUS staining and fluorometric determination. These experiments were done three times.

### 4.9. Quantitative Real-Time PCR

Total RNA isolation and first-strand cDNA synthesis were conducted as described by Xia et al. [36]. The transcripts of stress-responsive genes were checked by quantitative real-time PCR (qPCR) using gene-specific primers (Table S2). The qPCR was performed in 96-well white plates containing 20 μL of reaction mixture, each in triplicate, on a IQ5 light cycler system (Bio-Rad, Hercules, CA, USA) as described by us [36]. Relative transcript levels of each gene were calculated according to the 2^−ΔΔCt^ method as described by Livaka and Schmittgen [44]. In these qPCR assays, *AtActin2* was used as the reference gene. All qPCR experiments were performed with three biological replicates and three technical replicates.

### 4.10. Statistical Analysis

All the results were expressed as mean values ± SD (standard deviation). For all the analyses, the statistical significance of the data was determined using Student′s *t*-test (*n* = 3, *p* < 0.05) at a 95% confidence level.

## Supplementary Materials

Supplementary materials can be found at https://www.mdpi.com/1422-0067/20/13/3326/s1.
