# Supplementary material for: Identification of a 119-bp Promoter of the Maize Sulfite Oxidase Gene (ZmSO) That Confers High-Level Gene Expression and ABA or Drought Inducibility in Transgenic Plants
Source: Int J Mol Sci. 2019 Jul 6;20(13):3326. doi: 10.3390/ijms20133326 (PMC6651508; doi:10.3390/ijms20133326)
Supplement: Supplementary file 1 [file ijms-20-03326-s001.pdf]

Supplementary Material

Table S1 Known *cis*-acting elements in the *pZmSO* using the PlantCARE and PLACE databases.

***Cis*-elements**	**Description**	**No**
CAAT-box	Common *cis*-acting element	17
TATA-box	Core promoter element	20
ABRE	*Cis*-acting element involved in the abscisic acid responsiveness	3
MBS	MYB binding site involved in drought-inducibility	2
TGACG-motif	*Cis*-acting regulatory element involved in the MeJA-responsiveness	2
G-box	*Cis*-acting regulatory element involved in light responsiveness	2
LTR	*C*is-acting element involved in low-temperature responsiveness	1
TGA-element	Auxin-responsive element	1

Table S2 PCR primers used for 5′ RACE-PCR, qPCR, and expression vectors of *ZmSO* promoter and its mutants in transgenic plants in this study.

**Primer name**	**Primer sequence*（5'--3'）**
SOP0-F	ACGAATTCCCGTGCTGTGCTGTCTGTCC
SOP1-F	ACGAATTCCCAATACAAG GGGTGTGTCAG
SOP2-F	ACGAATTCCCTGTACCTGTCTAACCAATGG
SOP3-F	AGGAATTCAATTATTCTATATAAAATC
SOP4-F	ACGAATTCTCGTCTCGTTCTCTTCTCCTCAC
Mutated SOP2-F1	TCGAATTCTAGAGAAATTATTCTATATAAAATCCGTA **GCTAGA**TTTAG
SOP-R	ACAAGCTTCTTCGCTAGCCTCCGCGGTGCG
Outer-specific primer	AATAGCCAAGAACCTCTATCC
Inner-specific primer	GGTGAGGCTCAGCATTGAATGG
RD29A-QF	CAGAGGAACCACCACTCAACACA
RD29A-QR	CTCTAGGTTTACCTGTTACGCCTG
DREB2A-QF	CGACTGTTGATTCTCTAT
DREB2A-QR	TTATTCATTCCTGTTGTTAC
Actin2-F	TTGTGCTGGATT CTGGTGATGG
Actin2-R	CCGCTCTGCTGTTGTGGTG

*The underlined nucleotides constitute *Eco*RI (GAATTC), or *Hin*dIII (AAGCTT) restriction enzyme digestion

sites. The nucleotides in bold indicate the mutated MYB binding site (MBS) sequence.

Fig.S1

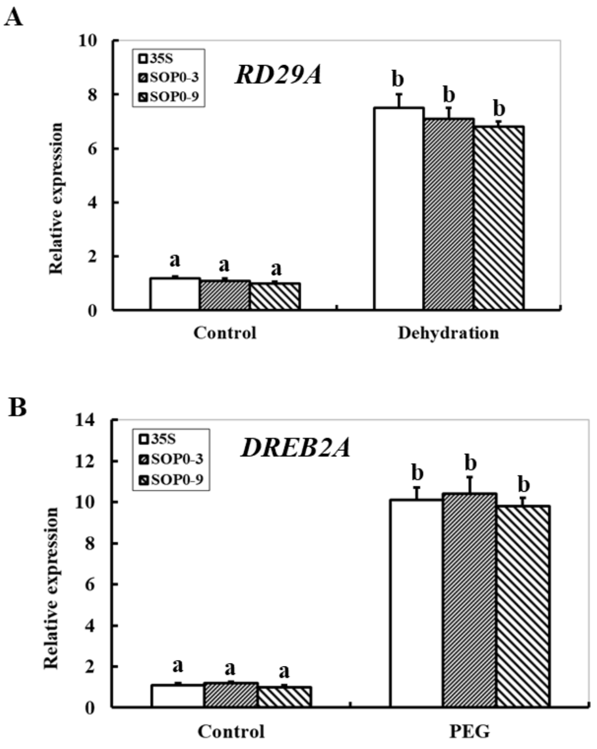

Figure S1. Expression of stress-induced marker genes in transgenic *Arabidopsis* expressing *ZmSOpro:GUS or CaMV* 35S in response to dehydration and osmotic stresses.

Two-week-old seedlings were incubated in the liquid MS medium supplemented with 10 % (w/v) PEG 6000 to realize osmotic stress for 6 h, or the seedlings were transferred onto filter papers to induce dehydration stress for 2 h. The seedlings grown in the liquid MS medium were treated as controls. After treatments, expression of stress-responsive genes *RD29A* (A) and *DREB2A* (B) was assayed by qPCR in seedlings of *CaMV* 35S and two *ZmSOpro:GUS* transgenic lines (SOP0-3 and -9). For each experiment, three technical replicates were conducted. Data shown are Mean ± SE of three independent experiments. Different lowercase letters above the bars indicate significant differences at $P < 0.05$.
